# Influence of Replacing Cement with Waste Glass on Mechanical Properties of Concrete

**DOI:** 10.3390/ma15217513

**Published:** 2022-10-26

**Authors:** Özer Zeybek, Yasin Onuralp Özkılıç, Memduh Karalar, Ali İhsan Çelik, Shaker Qaidi, Jawad Ahmad, Dumitru Doru Burduhos-Nergis, Diana Petronela Burduhos-Nergis

**Affiliations:** 1 Department of Civil Engineering, Faculty of Engineering, Mugla Sitki Kocman University, Mugla 48000, Turkey; 2 Department of Civil Engineering, Faculty of Engineering, Necmettin Erbakan University, Konya 42000, Turkey; 3 Department of Civil Engineering, Faculty of Engineering, Zonguldak Bulent Ecevit University, Zonguldak 67100, Turkey; 4 Department of Construction, Tomarza Mustafa Akincioglu Vocational School, Kayseri University, Kayseri 38940, Turkey; 5 Department of Civil Engineering, College of Engineering, University of Duhok, Duhok 42001, Iraq; 6 Department of Civil Engineering, College of Engineering, Nawroz University, Duhok 42001, Iraq; 7 Department of Civil Engineering, Military College of Engineering (NUST), Risalpur 24080, Pakistan; 8 Faculty of Materials Science and Engineering, Gheorghe Asachi Technical University of Iasi, 700050 Iasi, Romania

**Keywords:** eco-friendly concrete, waste glass, workability, compressive strength, splitting tensile strength, flexural strength

## Abstract

In this study, the effect of waste glass on the mechanical properties of concrete was examined by conducting a series of compressive strength, splitting tensile strength and flexural strength tests. According to this aim, waste glass powder (WGP) was first used as a partial replacement for cement and six different ratios of WGP were utilized in concrete production: 0%, 10%, 20%, 30%, 40%, and 50%. To examine the combined effect of different ratios of WGP on concrete performance, mixed samples (10%, 20%, 30%) were then prepared by replacing cement, and fine and coarse aggregates with both WGP and crashed glass particles. Workability and slump values of concrete produced with different amounts of waste glass were determined on the fresh state of concrete, and these properties were compared with those of plain concrete. For the hardened concrete, 150 mm × 150 mm × 150 mm cubic specimens and cylindrical specimens with a diameter of 100 mm and a height of 200 mm were tested to identify the compressive strength and splitting tensile strength of the concrete produced with waste glass. Next, a three-point bending test was carried out on samples with dimensions of 100 × 100 × 400 mm, and a span length of 300 mm to obtain the flexure behavior of different mixtures. According to the results obtained, a 20% substitution of WGP as cement can be considered the optimum dose. On the other hand, for concrete produced with combined WGP and crashed glass particles, mechanical properties increased up to a certain limit and then decreased owing to poor workability. Thus, 10% can be considered the optimum replacement level, as combined waste glass shows considerably higher strength and better workability properties. Furthermore, scanning electron microscope (SEM) analysis was performed to investigate the microstructure of the composition. Good adhesion was observed between the waste glass and cementitious concrete. Lastly, practical empirical equations have been developed to determine the compressive strength, splitting tensile strength, and flexure strength of concrete with different amounts of waste glass. Instead of conducting an experiment, these strength values of the concrete produced with glass powder can be easily estimated at the design stage with the help of proposed expressions.

## 1. Introduction

The use of ordinary Portland cement (OPC) by replacing it with recycled cement in certain proportions is an interesting issue for sustainable environmental awareness [1,2]. During cement production, a high amount of energy is consumed, and a high amount of carbon dioxide (CaO_2_) is released into the atmosphere [3]. Therefore, cement additives are of interest to reduce cement production [4,5]. Waste is generally defined as residues from industrial production processes, residues arising from the transfer, or product residues that have completed their economic life [6,7,8,9,10,11,12,13,14]. With the development of modern cities, the decrease in natural resources, climate change, and increasing awareness of environmental protection, there is an urgent need to develop building materials that will reduce greenhouse gas emissions [2,15]. Waste glass powder (WGP), which is increasing with the effect of industrialization and the increase in urban transformation, attracts the attention of researchers as a concrete additive material due to its economic and mechanical performance effects [4,5,16,17,18,19,20,21]. Since there is no regular storage area for waste glass, it increases the risk of soil and water pollution due to its oxidation effect. Therefore, the use of recycled glass in concrete production will provide significant contributions to reducing environmental problems [2,3,22,23].

According to ASTM C618-19, 2019, since waste glass consists of a large amount of calcium and an amorphous structure, it can be ground into powder to obtain pozzolanic material or cement additive [24,25,26]. Therefore, WGP can be used in concrete production by replacing it with cement in certain proportions [2]. Recent studies show that replacing 15–25% glass powder with cement increases the mechanical properties of concrete [6,15,21]. Aliabdo et al. used 25% substitution with cement to investigate the mechanical effect of WGP in concrete as a cement substitute. According to the results obtained, it was observed that the void ratio and density of the samples decreased, while the tensile and compressive strengths increased [27]. Al Saffar et al. in their study to test the interaction of WGP, added cement mortar with other materials and found that the compressive strength of the samples increased as the glass powder addition rate increased. They obtained the highest strength with the addition of 25% glass powder [15]. Elaqra et al. (2019) added 4% by weight to the mix to investigate the effect of WGP on fresh and hardened concrete. They found that as the amount of WGP increased, the machinability increased, and the maximum compressive strength was reached with the addition of 20% WGP at 28 days of cure [28]. It is well known that in the case of fine grinding of soda–lime glass, the reactivity of pozzolanic increases as the particle size decreases [29]. Zhang et al. in their study to examine the effect of WGP particle size on the mechanical and microstructures of concrete, found that the particle size distribution of WGP has a significant effect on the properties of WGP-based concrete [30]. Shao et al. in their experimental study to observe the effect of finely ground WGP on the compressive strength of concrete found that the pozzolanic reactivity increases as the WGP particle size decreases. They found that WGP, with a particle size of 38 µm, was replaced by 30% with cement, resulting in 4.1 MPa greater compressive strength [31]. It was found that WGP pozzolanic reactivity was replaced by 0, 15, 30, 45, and 60% of weight cement, while below 30% the concrete compressive strength did not decrease due to the pozzolanic reaction between WGP cement hydration products. In fact, with the addition of 60% WGP, the resistance to chloride ion and water penetration increased continuously, while the concrete compressive strength increased by around 85% [32]. In the study by Peril and Sangle, it was observed that when WGP was mixed with 30% cement, an increase in compressive strength between <38 µm and <75 µm was between 20% and 10% [33]. Khatib et al. found in their study that there was a 1.2% increase in concrete compressive strength with the addition of 10% WGP [34]. In a similar study, Madandoust and Ghavidel found that the compressive strength of the control sample was higher than the concrete with glass powder added at every stage, the compressive strength of both samples increased with aging [35]. In their study, Tejaswi et al. stated that the compressive strength increased by 1.5% with the addition of 10% WGP. However, they found that the compressive strengths were at equal levels when 20% was substituted [36]. Vasudevan et al. observed a 1.05% increase in concrete compressive strength when 20% WFP addition was <90 µm [37]. Schwarz et al. found that the addition of 20% WGP increased the compressive strength by 19% when <75 µm [38]. They state that while the strength decreases as a result of the 7-day compressive strength test, the compressive strength does not change as a result of the 28- and 91-day tests <75 µm. In addition to strength performance, the failure processes of brittleness materials are also important. Some researchers have studied the failure processes of brittleness materials [39,40]. Since the risk of fracture will increase under sudden loads, it is beneficial to take measures to increase elastic behavior.

Dust and particles from soda glass were mostly used in previous studies. However, its usability in concrete was investigated by replacing fine aggregate in self-compacting concrete with waste particles obtained from cathode ray tubes. The results had a positive effect on the durability properties of concrete [41]. Past studies have shown that ground glass powder can increase the pozzolanic reactivity of secondary cementitious materials. Therefore, the granule size of the ground glass powder has important effects [42,43]. Ahmet et al. examined different methods of using waste glass in concrete and stated that particle size, substitution ratio, and chemical composition have important effects on the mechanical durability of concrete. For example, as the grain size decreases, workability becomes more difficult but pozzolanic and strength increase [44]. The particle size of the waste glass, which must be taken into account during the mix design, may affect the active silica reaction depending on the rate of substitution.

Solid waste management is an important issue for most developing countries [45]. Instead of storing or disposing of waste materials such as glass, plastic, and metal, reusing or recycling has become a more attractive option. Waste glass has started to be widely preferred for concrete production in civil engineering applications in recent years. Since the employing of waste glass in concrete can assist to reduce environmental pollution, protect natural resources and produce low-cost concrete, WGP can be preferred instead of either natural aggregates or cement due to its pozzolanic effect. Although many studies have been conducted on this subject, there are differences in the results obtained from the literature. Thus, there still remains a need to investigate the mechanical behavior of concrete with partial substitution of waste glass and the ideal dosage of it. Based on this motivation, an experimental study was carried out on some test specimens. Analytical solution proposals have been developed according to the data obtained from the experimental study. The proposed formulas will serve as a guide for researchers and manufacturers and will accelerate future studies to be more effective.

## 2. Experimental Program

**Figure 1:**
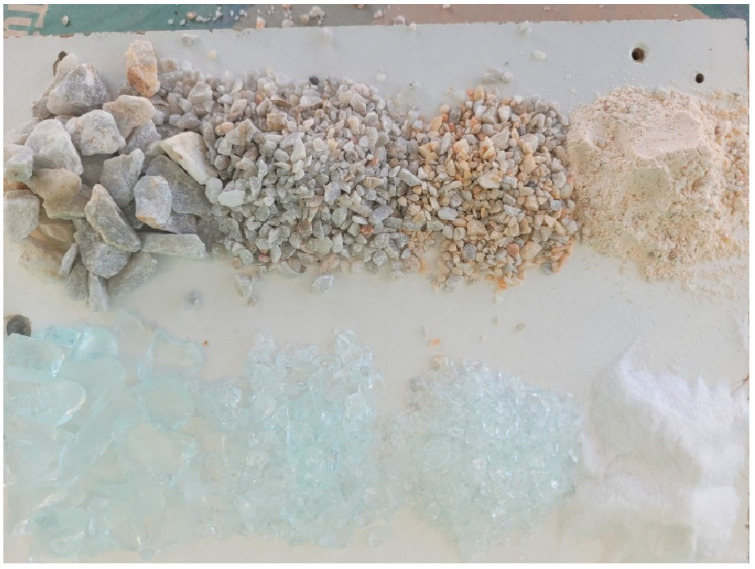
Recycled glass, cement, and aggregates.

**Figure 2:**
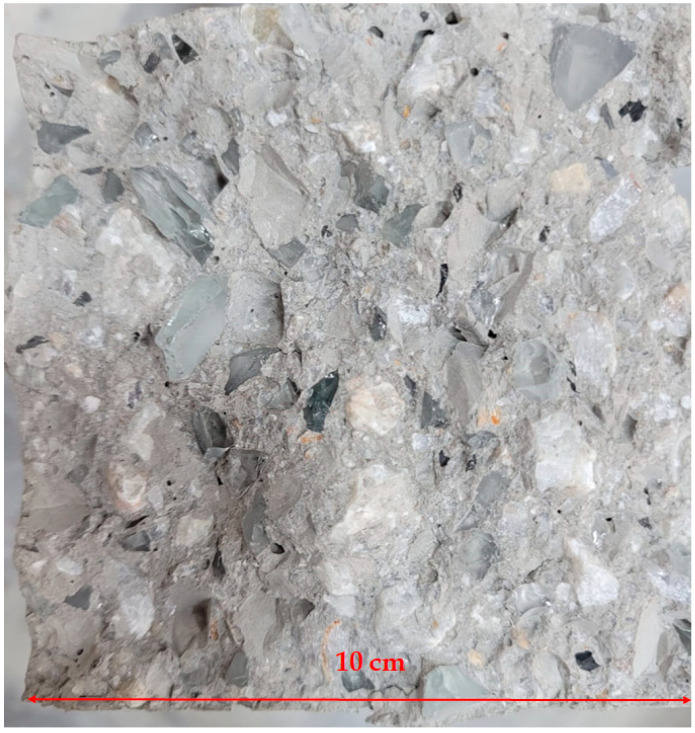
Recycled glass in concrete.

In this study, the main aim is to investigate the effect of glass powder when it is replaced with cement. Furthermore, the effects of all replacements with glass are investigated. Nine mixes including the reference were designed. C represents cement replacement, MIX represents cement, fine aggregate, and coarse aggregate replacement. Table 1 summarizes the sample properties. For the MIX design, each type of material was replaced with certain amounts of recycled glass. The size of fine aggregates is 1 mm to 4 mm and the corresponding waste glass was 1.7 mm to 4 mm. Size of coarse aggregate size was selected as 5–12 mm and the corresponding waste glass was also 5–12 mm. The particle size of cement particles was between 0.02 mm to 0.1 mm and the size of glass waste powder was 0.1–0.2 mm. Figure 1 demonstrates the used aggregates and cement and also their replacements which are glass powder and glass grains. Figure 2 demonstrates the recycled glass in concrete.

**Figure 3:**
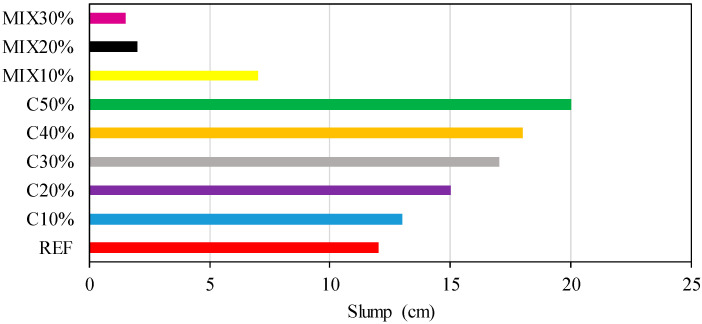
Slump test.

Cement was selected as CEM I 32.5 Portland cement. The water-to-cement ratio was chosen as 0.5. Figure 3 demonstrates the slump test results. The workability of the concrete produced by adding WGP increased as the waste rate increased. Accordingly, there was an increase in slump values. It is seen that the slump value increases as the recycled glass ratio increases. On the other hand, when all ingredients were replaced with glass, the slump value significantly decreases. Madandous and Ghavidel obtained an 80 mm slump in their study with the addition of 5–20% WGP. In this study, it is seen that the slump value is 200 mm, since the additional WCP is 50%. It can be said that the results are similar to the literature [35].

Three types of tests were performed in order to evaluate the performance of the concrete with recycled glass. A compression test was performed on 15 × 15 × 15 cm cubic samples while splitting tests were conducted on 10 × 20 cm cylinder samples. Furthermore, bending tests were performed using 10 × 10 × 40 cm samples. Three repetitions were completed for each mix and each test. Compressive ability is the capability of a specimen to decline load under pressure. The CST was performed according to ASTM C39/C39M (C39&C39M A, 2003). In this test, specimens have dimensions of 150 mm × 300 mm. In this experiment, concrete specimens were subjected to constraint lengthwise load at a level in the offered as concerns the specimen fractures. At that point, the compressive capability was estimated from the critical failure strength separated by the part of the specimen. To provide the tensile strength of concrete where compactor load is performed in anticipation of examples unravel caused by expansion of tensile force in concrete as ASTM (Designation, 1976). In this way, cylindrical examples are divided through the vertical dimension.

## 3. Experimental Results and Discussions

### 3.1. Compressive Strength (CS)

**Figure 4:**
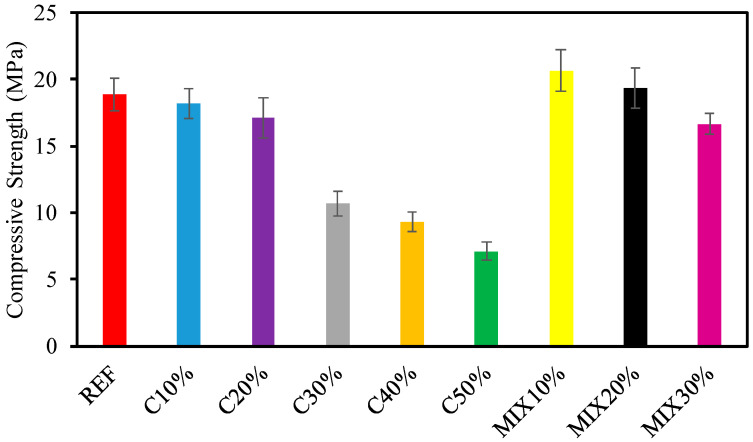
Results of CS.

In this part, to carry through the compressive strength test (CST), concrete examples were arranged without any adulteration. Figure 4 expresses the CST consequences at 28 days period for low w/c ratio combinations formed with 100% reference and numerous ratios of recycled aggregate substituting reference aggregates, with and lacking waste glass. The CS values of the reference concrete without waste glass series at 28 days were found as 18.9 MPa, respectively. The lowest CS values were also found to be 7.10 MPa in the concrete produced with cement including 50% waste glass for 28 days periods, respectively. As observed in Figure 4, it has been detected that the CS of concrete combinations including waste glass utilization as a fractional replacement for cement were lesser than those of the corresponding concrete mixes lacking waste glass. As observed in Figure 4, statistical investigation of test values shows the noteworthy impact of waste glass (as a fractional replacement for cement) on the CS of concrete.

In the comparison of the cement that was changed with waste glass powder, the CS of C10% (10% cement replaced with waste glass) is 6% greater than that of C20%. Correspondingly, the CS of C20% (20% cement replaced with waste glass) is 60% greater than that of C30%. Comparing the CS of C30% (30% of the cement was replaced with waste glass powder) it can be observed that the value is 15% greater than that of C40%. Finally, the comparison of CS of C40% (40% of the cement was substituted with waste glass powder) is 31% greater than that of C50%. Consequently, it is observed that the CS of C10% (10% cement was replaced with waste glass powder) is similar to that of reference concrete. As shown in Figure 4, while fine and coarse aggregate for 10%, 20%, and 30% (MIX10%, MIX20%, and MIX30%) were exchanged with waste glass, this trend was reversed as fractional replacement of cement with waste glass promoted the CS of concrete. On the other hand, at the comparison of CS of MIX10%, MIX20%, and MIX30%, the CST consequences for the great waste glass ratio continue trends comparable to those for the small waste glass ratio. In this situation too, the CS of MIX10% is 24% greater than the corresponding mix (MIX30%). While waste glass is used instead of cement, fine and coarse aggregate, remarkable improvements in strength are observed when compared with the reference concrete. Therefore, it was observed that by replacing cement with glass powder, a significant reduction in CS will be obtained.

### 3.2. Splitting Tensile Strength (STS)

**Figure 5:**
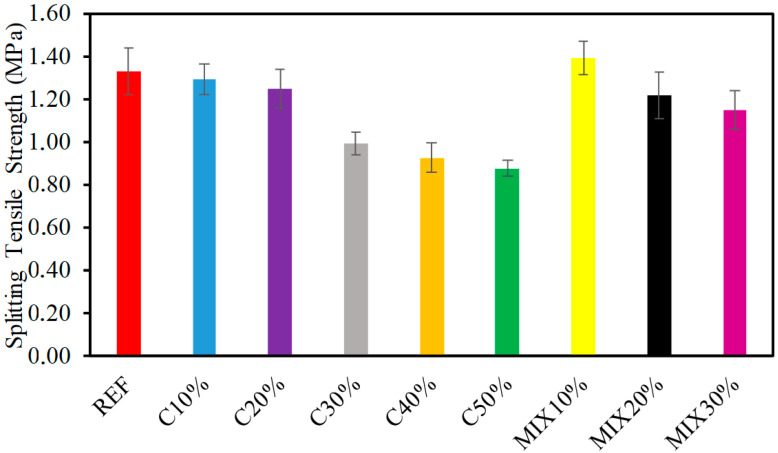
Results of STS.

**Figure 6:**
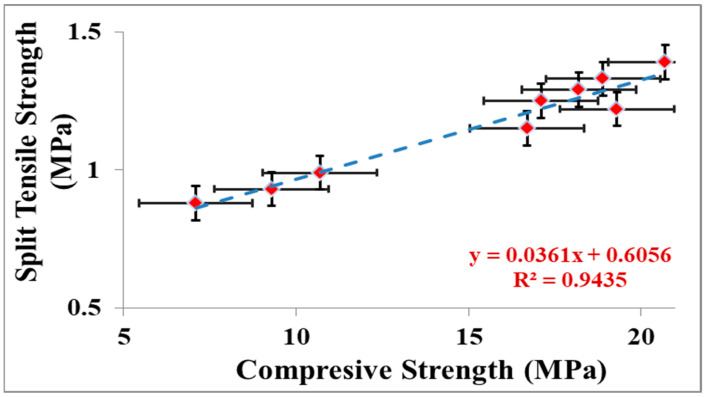
STS versus CS.

A comparison of relative STS of the various concrete percentage combinations formed with 100% reference and numerous ratios of recycled aggregate replacing reference aggregates, with and without waste glass are shown in Figure 5. As presented in Figure 5, the results of STS commonly pursue a similar tendency as the compressive strength. As observed from Figure 5, the remarkable improvement in concrete tensile strength is up to 10% with waste glass used as a fractional replacement for cement. Upon altering the amount of waste glass, consequences of tensile strength are influenced similarly to the CS. As shown in Figure 5, tensile strength optimal norms are found at 10% waste glass obligating maximum divided tensile. A comparative evaluation was performed where controller concrete tensile strength was deliberate as reference strength, from which concrete of the changing ratio of waste glass is collated. At 10% substitution of waste glass, STS is only 3% lower than the reference mix. The correlation of CS in competition with STS is provided in Figure 6. As shown in Figure 6, the regression model among the compressive and STS appeared to be flat. Furthermore, as observed in Figure 6, the regression stroke represents a strong relationship between CS contrasted with STS having an R2 worth of more than 94 percent.

### 3.3. Flexural Performance

**Figure 7:**
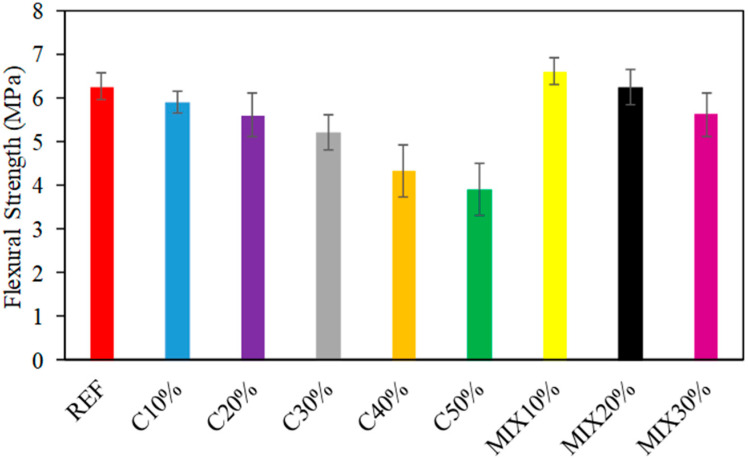
Results of FS.

**Figure 8:**
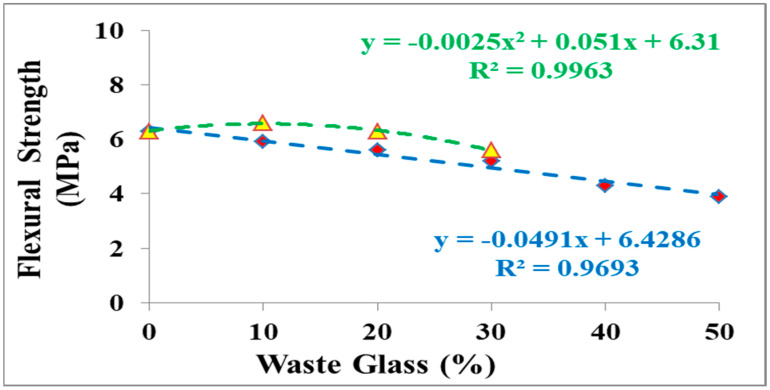
Results of tensile flexural strength tests of concrete examples with altered waste glass substances.

Flexure strength (FS) of investigational examples was established on examples after CST. The acquired norms of strength ranged between 6.6 MPa and 3.9 MPa. The consequence of the FS of the examples is presented in Figure 7. In Figure 7, it is detected FS with variable ratios of waste glass. Related to CS, FS at the initial phase failures with the incorporation of waste glass. It was detected that with the addition of waste glass at 10%, 20%, 30%, 40%, and 50% of cement weight, the decrease in FS was 6.7%, 12.5%, and 21.1%, 46.5%, and 61.5% correspondingly in proportion to the reference sample (6.3 MPa). Figure 8 demonstrates a rectilinear relationship between the tensile FS of the example and the substance of waste glass addition. On the other hand, if fine and coarse aggregates in cement are replaced with waste glass, it was noticed that the waste glass was replaced with the cement, fine and coarse aggregate at 10%, the increase in FS was 4.7% correspondingly in proportion to the reference sample (6.3 MPa). Nevertheless, if the quantity of waste glass is occupied relative to the cement content, it may be detected that the association between FS and waste glass content is related (the slope of the curves is approximate (Figure 8)).

### 3.4. Comparison of Findings of this Study with Other Existing Studies

**Figure 9:**
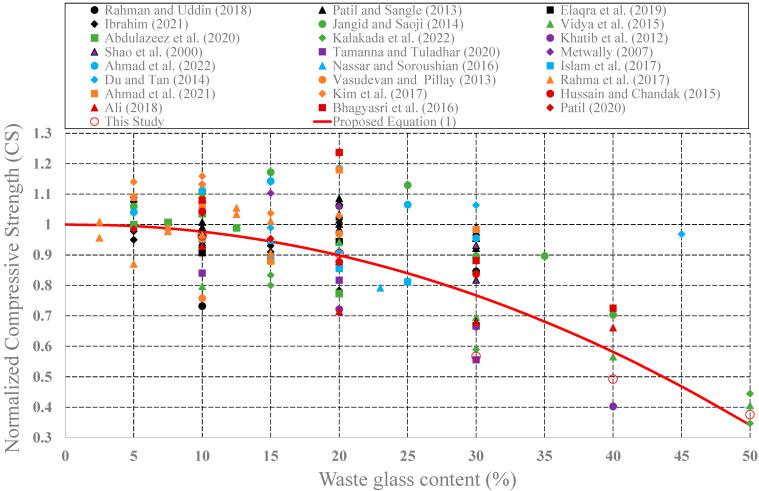
Variation of the normalized CS of the concrete produced with waste glass.

**Figure 10:**
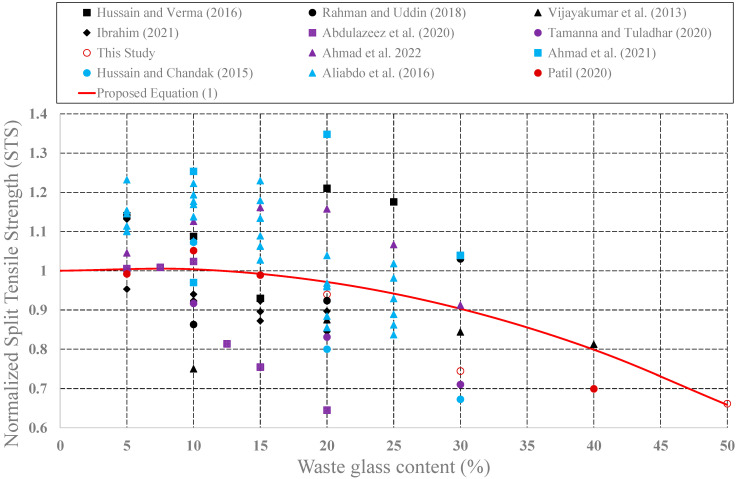
Variation of the STS of the concrete produced with waste glass.

**Figure 11:**
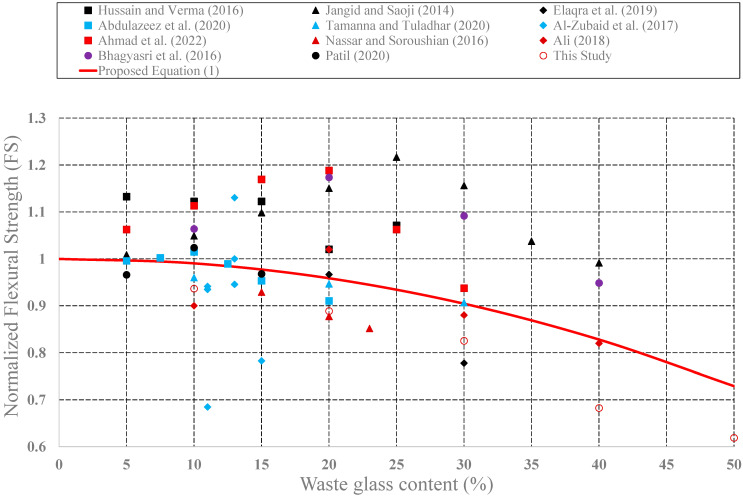
Variation of the FS of the concrete produced with waste glass.

The effect of waste glass on the structural performance of concrete was investigated by many research teams. Effects of the use of waste glass as cement replacement on some mechanical properties such as CS, STS, and FS have been investigated. In this part of the study, values of CS, STS, and FS for plain concrete and concrete produced from different glass powder amounts have been collected from the research publications [46,47,48,49,50,51,52,53,54,55,56,57,58,59,60,61,62,63]. Then, measured strength values of concrete produced with waste glass were first normalized by plain concrete strengths. These normalized strength values were plotted in Figure 9, Figure 10 and Figure 11, respectively, as a function of the waste glass content.

As shown in these figures, with the addition of waste glass, the strength values of concrete produced with glass powder generally decrease after a certain value. The maximum decrease in compressive strength was observed in the study by Kalakada et. al., 2022. When the cement was partially replaced with glass powder at 50%, there is a 65% reduction in compression strength of the plain concrete specimen. In the same manner, a 20% addition of the waste glass leads to a 36% reduction in the splitting tensile strength of the plain concrete specimen in the study of Abdulazeez et al., (2020). With the addition of 50% glass powder, a 38% reduction in flexural strength was observed in our experimental study. Thus, a rational design expression was developed to consider these reductions in the strength values as follows:

(1) $$f=[1+c_{1}\times \left(WGPR\right)+c_{2}\times {\left(WGPR\right)}^{2}]\times f\prime$$

where *f* is strength values as follows: *f_c_*: compressive strength *f_t_*: splitting tensile strength; *f_f_*: flexure strength; *WGPR*: waste glass power ratio (0 < *WGPR* < 50). *c_1_* and *c_2_* are coefficients given in Table 2; *f*′ is strength values of the plain concrete.

As shown in Equation (1), CS, STS, and FS values of concrete produced with waste glass were expressed as a function of the amount of the waste glass (*WGPR*). These expressions can be easily used in the design stages.

### 3.5. Scanning Electron Microscope (SEM) Analysis

**Figure 12:**
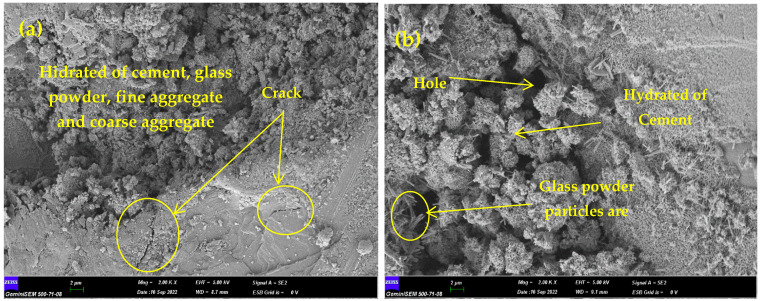

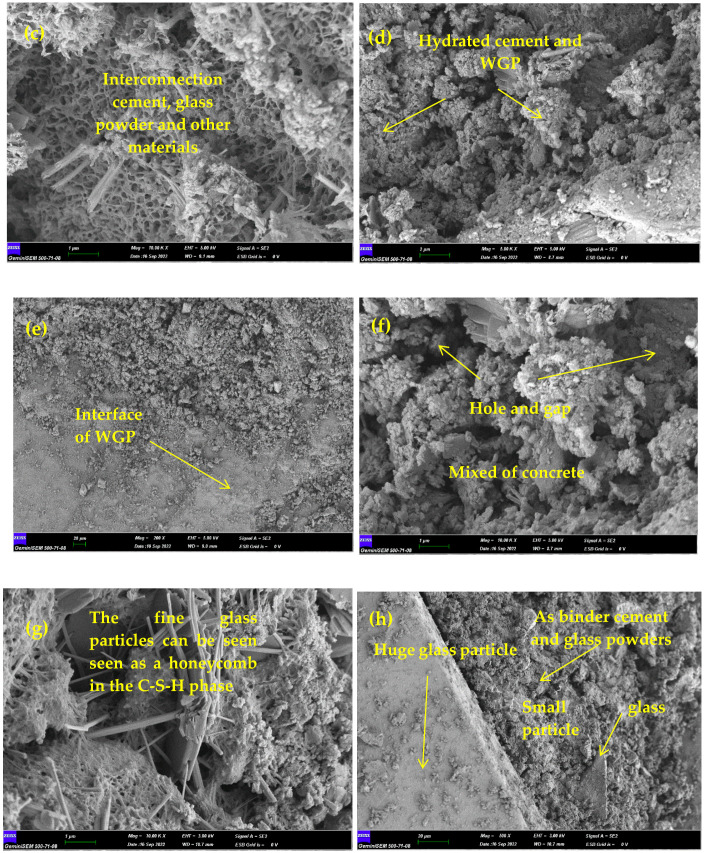

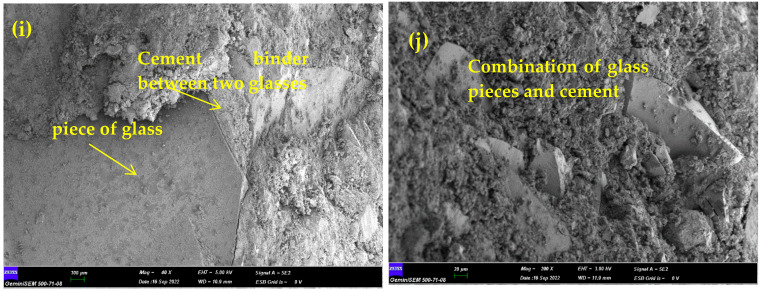
SEM micrographs of the obtained samples.

Scanning electron microscope (SEM) analysis was performed from the sample pieces taken after the compressive strength test from the concrete samples produced with recycled glass waste powder. SEM analysis is carried out to show the typical morphology of the surface tissue of OPC and WGP. The particles of both OPC and WGP are composed of glassy structures and irregular shapes with sharp edges. OPC particles consist of sharper edges and shapes, while WGP particle appears on smoother surfaces, sharper prismatic edges, and denser content [2]. The best images were selected to observe the effect of glass dust and glass particles in OPC concrete production. Figure 12a–f contains images where WGP is replaced by cement. Images of the mix design samples from Figure 12g–j contain the details of the change in glass particles with cement and aggregates. In order for WGP to be processed as a filler by grinding in electric mills without any treatment, 74% of the particles must be passed through a 36 µm sieve [64]. The key findings of the inner image details at 500 times magnification are shown in detail in Figure 12. Judging from the general view in Figure 12a, it can be said that glass powder provides good bonding with cement and aggregates. A good interlocking is observed in terms of surface quality, but some flat zones have cracks due to fracture. In Figure 12b, the appearance of glass powder particles like sharp fibers is remarkable. Although there is a homogeneous distribution, gaps are seen in some regions. Voids can be removed by better mixing, shaking, and skewering [64,65,66]. The image in Figure 12c shows material connections more closely, such as a finely woven bee comb. Figure 12d shows the concrete surface bonding and spacing. Figure 12e,f shows the gelled state of the concrete surface. In terms of surface quality, it can be said that the glass powder is in a good correlation, but it has some holes and gaps. In the mixed model, glass powder was used as a binder with cement, and broken glass particles were used by replacing fine and coarse aggregates in certain proportions. Calcium silicate hydrate (C-S-H), an amorphous phase, is defined as weak crystals [67]. The fine glass particles can be seen in Figure 12g. They are seen as a honeycomb in the C-S-H phase. Since this honeycomb structure provides a good pozzolanic effect, it increases compressive strength [68,69]. Figure 12h shows how large and small glass particles are bonded with binders in concrete. It is seen in Figure 12i with good fillings of binders between large pieces of glass. Figure 12j shows the homogeneous distribution and connection of the glass particles in the concrete.

## 4. Conclusions and Summary

Waste management has gained great importance due to the increase in the amount of waste. The reuse or recycling approach of these waste products, especially in the construction sector, has become a more attractive option. In this study, the applicability of waste glass on concrete production was investigated by considering two different conditions, i.e., replacing cement with waste glass powder and replacing cement, fine and coarse aggregate with waste glasses. A series of tests were conducted on concrete samples produced with different amounts of waste glass to determine engineering properties both in the fresh and hardened cases. In the fresh case of concrete, workability and slump properties were explored. In the case of hardened concrete, compression strength, splitting tensile strength, and flexural strength of the produced test specimens were investigated. These properties were then compared with those of plain concrete. Then, SEM analysis was carried out to explore the interaction between glass powder, cement, and aggregates. Furthermore, practical equations were developed to identify the compressive strength, splitting tensile strength, and flexure strength of concrete produced with WGP. Based on our study, the following conclusions can be drawn:

- Based on the slump test, the slump value decreases as the amount of waste glass increases. In the same manner, the workability decreases when the rate of glass powder increases.
- The compressive test results indicated that the usage of WGP as a replacement of cement adversely affects the compressive strength of concrete. Compared to the reference sample, 10%, 20%, 30%, 40% and 50% replacement of WPG reduced the compressive strength by 3%, 6%, 37%, 13% and 23%, respectively.
- On the other hand, when cement, fine and coarse aggregates were replaced with waste glasses, there was an increase in the compression strength, the flexural and splitting tensile strength values up to a certain value of the amount of the waste. However, further increasing the waste glass addition led to a decrease in the compression value.
- According to the results of the split tensile and bending strength tests, the strength values decrease as the value of the substitution of cement with waste glass increases. Compared to plain concrete, 50% replacement of WPG reduced the splitting tensile strength by 34%, respectively. On the other hand, this replacement reduced flexural strength by 38%.
- When cement, fine and coarse aggregates were replaced with waste glasses there was an increase in the flexural and splitting tensile strength values up to a certain value of the amount of the waste glass. However, further increasing the waste glass addition resulted in a decrease in the strength values. Replacing the waste glass by 10% resulted in a 5% increase in splitting tensile strength while replacing the waste glass by 30% resulted in a 14% decrease in splitting tensile strength. On the other hand, a 10% substitute of waste glass resulted in a 5% increase in flexural strength, while a 30% substitute of waste glass resulted in an 11% decrease in flexural strength.
- It can be concluded that when waste glass is used instead of cement, fine and coarse aggregate, significant increases in strength are observed.
- SEM analysis results showed that there was a good bond between the glass powder and cementitious concrete.
- The proposed equations for the compressive strength, flexural, and splitting tensile strength are quite general and they can be suitable for direct adoption into design specifications of the concretes produced with waste glass.

In this study, it was observed that waste glass can be used as a partial cement substitute, or as cement, fine and coarse aggregate in concrete. Optimum waste glass dosage has also been identified for design purposes. However, lower strength values were observed when waste glass was used as a partial replacement for cement. The current study defines waste glass as suitable, reachable in large amounts, a local eco-material, cheap, that can be selected for concrete construction, in a point of view between economically and environmentally responsive. To mitigate this shortcoming, future studies will concentrate on the use of waste glass with other recycling fibers.

## Figures and Tables

**Table 1:** Sample Properties.

REF	No Glass Was Used
C10%	10% cement was replaced with waste glass powder
C20%	20% cement was replaced with waste glass powder
C30%	30% cement was replaced with waste glass powder
C40%	40% cement was replaced with waste glass powder
C50%	50% cement was replaced with waste glass powder
MIX10%	10% cement, 10% fine and 10% coarse aggregates were replaced with waste glass
MIX20%	20% cement, 20% fine and 20% coarse aggregates were replaced with waste glass
MIX30%	30% cement, 30% fine and 30% coarse aggregates were replaced with waste glass

**Table 2:** Constants for Equation (1).

Strength Values (*f*)	*c_1_*	*c_2_*
*f_c_*	0.00033	−0.00027
*f_t_*	0.00217	−0.00018
*f_f_*	0.00017	−0.00011

## Data Availability

Not applicable.

## References

[1] Batayneh M., Marie I., Asi I. (2007). Use of selected waste materials in concrete mixes. Waste Manag..

[2] Jiang X., Xiao R., Bai Y., Huang B., Ma Y. (2022). Influence of waste glass powder as a supplementary cementitious material (SCM) on physical and mechanical properties of cement paste under high temperatures. J. Clean. Prod..

[3] Bilondi M.P., Toufigh M.M., Toufigh V. (2018). Experimental investigation of using a recycled glass powder-based geopolymer to improve the mechanical behavior of clay soils. Constr. Build. Mater..

[4] Tayeh B.A., Al Saffar D.M., Aadi A.S., Almeshal I. (2020). Sulphate resistance of cement mortar contains glass powder. J. King Saud. Univ. Eng. Sci..

[5] Tayeh B.A., Almeshal I., Magbool H.M., Alabduljabbar H., Alyousef R. (2021). Performance of sustainable concrete containing different types of recycled plastic. J. Clean. Prod..

[6] Wang H.-Y., Huang W.-L. (2010). Durability of self-consolidating concrete using waste LCD glass. Constr. Build. Mater..

[7] Wang H.-Y., Zeng H.-H., Wu J.-Y. (2014). A study on the macro and micro properties of concrete with LCD glass. Constr. Build. Mater..

[8] Aksoylu C., Özkılıç Y.O., Hadzima-Nyarko M., Işık E., Arslan M.H. (2022). Investigation on Improvement in Shear Performance of Reinforced-Concrete Beams Produced with Recycled Steel Wires from Waste Tires. Sustainability.

[9] Ali E.E., Al-Tersawy S.H. (2012). Recycled glass as a partial replacement for fine aggregate in self compacting concrete. Constr. Build. Mater..

[10] Qaidi S., Tayeh B.A., Zeyad A.M., de Azevedo A.R., Ahmed H.U., Emad W. (2022). Recycling of mine tailings for the geopolymers production: A systematic review. Case Stud. Constr. Mater..

[11] Ahmed S.N., Sor N.H., Ahmed M.A., Qaidi S.M. (2022). Thermal conductivity and hardened behavior of eco-friendly concrete incorporating waste polypropylene as fine aggregate. Mater. Today Proc..

[12] Ahmad J., Martínez-García R., De-Prado-Gil J., Irshad K., El-Shorbagy M.A., Fediuk R., Vatin N.I. (2022). Concrete with Partial Substitution of Waste Glass and Recycled Concrete Aggregate. Materials.

[13] Qaidi S., Najm H.M., Abed S.M., Özkılıç Y.O., Al Dughaishi H., Alosta M., Sabri M.M.S., Alkhatib F., Milad A. (2022). Concrete Containing Waste Glass as an Environmentally Friendly Aggregate: A Review on Fresh and Mechanical Characteristics. Materials.

[14] Karalar M., Özkılıç Y.O., Deifalla A.F., Aksoylu C., Arslan M.H., Ahmad M., Sabri M.M.S. (2022). Improvement in Bending Performance of Reinforced Concrete Beams Produced with Waste Lathe Scraps. Sustainability.

[15] Giannopoulou I., Dimas D., Maragkos I., Panias D. (2009). Utilization of metallurgical solid by-products for the development of inorganic polymeric construction materials. Glob. NEST J..

[16] Al Saffar D.M.A.R. (2017). Experimental investigation of using ultra-fine glass powder in concrete. Int. J. Eng. Res. Appl..

[17] Almeshal I., Al-Tayeb M.M., Qaidi S.M., Abu Bakar B., Tayeh B.A. (2022). Mechanical properties of eco-friendly cements-based glass powder in aggressive medium. Mater. Today: Proc..

[18] Ez-Zaki H., El Gharbi B., Diouri A. (2018). Development of eco-friendly mortars incorporating glass and shell powders. Constr. Build. Mater..

[19] Jubeh A.I., Al Saffar D.M., Tayeh B.A. (2019). Effect of recycled glass powder on properties of cementitious materials contains styrene butadiene rubber. Arab. J. Geosci..

[20] Mousa M., Cuenca E., Ferrara L., Roy N., Tagnit-Hamou A. Tensile Characterization of an “Eco-Friendly” UHPFRC with Waste Glass Powder and Glass Sand. Proceedings of the International Conference on Strain-Hardening Cement-Based Composites.

[21] Tayeh B.A. (2018). Effects of marble, timber, and glass powder as partial replacements for cement. J. Civ. Eng. Constr..

[22] Tan K.H., Du H. (2013). Use of waste glass as sand in mortar: Part I—Fresh, mechanical and durability properties. Cem. Concr. Compos..

[23] Xiao R., Polaczyk P., Zhang M., Jiang X., Zhang Y., Huang B., Hu W. (2020). Evaluation of Glass Powder-Based Geopolymer Stabilized Road Bases Containing Recycled Waste Glass Aggregate. Transp. Res. Rec. J. Transp. Res. Board.

[24] (2013). Committee Specification for Coal Fly Ash and Raw or Calcined Natural Pozzolan for Use in Concrete.

[25] Omran A., Tagnit-Hamou A. (2016). Performance of glass-powder concrete in field applications. Constr. Build. Mater..

[26] Vijayakumar G., Vishaliny H., Govindarajulu D. (2013). Studies on glass powder as partial replacement of cement in concrete production. Int. J. Emerg. Technol. Adv. Eng..

[27] Aliabdo A.A., Abd Elmoaty A.E.M., Aboshama A.Y. (2016). Utilization of waste glass powder in the production of cement and concrete. Constr. Build. Mater..

[28] Elaqra H.A., Haloub M.A.A., Rustom R. (2019). Effect of new mixing method of glass powder as cement replacement on mechanical behavior of concrete. Constr. Build. Mater..

[29] Zheng K. (2016). Pozzolanic reaction of glass powder and its role in controlling alkali–silica reaction. Cem. Concr. Compos..

[30] Zhang Y., Xiao R., Jiang X., Li W., Zhu X., Huang B. (2020). Effect of particle size and curing temperature on mechanical and microstructural properties of waste glass-slag-based and waste glass-fly ash-based geopolymers. J. Clean. Prod..

[31] Shao Y., Lefort T., Moras S., Rodriguez D. (2000). Studies on concrete containing ground waste glass. Cem. Concr. Res..

[32] Du H., Tan K.H. (2014). Waste Glass Powder as Cement Replacement in Concrete. J. Adv. Concr. Technol..

[33] Patil D.M., Sangle K.K. (2013). Experimental investigation of waste glass powder as partial replacement of cement in concrete. Int. J. Adv. Technol. Civ. Eng..

[34] Khatib J.M., Negim E.M., Sohl H.S., Chileshe N. (2012). Glass powder utilisation in concrete production. Eur. J. Appl. Sci..

[35] Madandoust R., Ghavidel R. (2013). Mechanical properties of concrete containing waste glass powder and rice husk ash. Biosyst. Eng..

[36] Tejaswi S.S., Rao R.C., Vidya B., Renuka J. (2015). Experimental investigation of waste glass powder as partial replacement of cement and sand in concrete. IUP J. Struct. Eng..

[37] Vasudevan G., Pillay S.G.K. (2013). Performance of using waste glass powder in concrete as replacement of cement. Am. J. Eng. Res..

[38] Schwarz N., Cam H., Neithalath N. (2008). Influence of a fine glass powder on the durability characteristics of concrete and its comparison to fly ash. Cem. Concr. Compos..

[39] Qiu H., Zhu Z., Wang F., Wang M., Zhou C., Luo C., Wang X., Mao H. (2020). Dynamic behavior of a running crack crossing mortar-rock interface under impacting load. Eng. Fract. Mech..

[40] Wang F., Wang M., Zhu Z., Deng J., Nezhad M.M., Qiu H., Ying P. (2020). Rock Dynamic Crack Propagation Behaviour and Determination Method with Improved Single Cleavage Semi-circle Specimen Under Impact Loads. Acta Mech. Solida Sin..

[41] Sua-Iam G., Makul N. (2012). Use of Limestone Powder to Improve the Properties of Self-Compacting Concrete Produced Using Cathode Ray Tube Waste as Fine Aggregate. Appl. Mech. Mater..

[42] Alexander K.M. (1960). Reactivity of ultrafine powders produced from siliceous rocks. J. Proc..

[43] Vizcayno C., DE Gutierrez R.M., Castello R., Rodriguez E., Guerrero C. (2010). Pozzolan obtained by mechanochemical and thermal treatments of kaolin. Appl. Clay Sci..

[44] Ahmad J., Zhou Z., Usanova K.I., Vatin N.I., El-Shorbagy M.A. (2022). A Step towards Concrete with Partial Substitution of Waste Glass (WG) in Concrete: A Review. Materials.

[45] Çelik A.I., Özkılıç Y.O., Zeybek Ö., Özdöner N., Tayeh B.A. (2022). Performance Assessment of Fiber-Reinforced Concrete Produced with Waste Lathe Fibers. Sustainability.

[46] Ahmad J., Aslam F., Martinez-Garcia R., De-Prado-Gil J., Qaidi S.M.A., Brahmia A. (2021). Effects of waste glass and waste marble on mechanical and durability performance of concrete. Sci. Rep..

[47] Rahma A., El Naber N., Ismail I.S. (2017). Effect of glass powder on the compression strength and the workability of concrete. Cogent Eng..

[48] AbdulAzeez A.S., Idi M.A., Kolawole M.A., Hamza B. (2020). Effect of Waste Glass Powder as A Pozzolanic Material in Concrete Production. Int. J. Eng. Res..

[49] Kim S.K., Kang S.T., Kim J.K., Jang I.Y. (2017). Effects of Particle Size and Cement Replacement of LCD Glass Powder in Concrete. Adv. Mater. Sci. Eng..

[50] Rahman S., Uddin M. (2018). Experimental Investigation of Concrete with Glass Powder as Partial Replacement of Cement. Civ. Eng. Arch..

[51] Jangid B.J., Saoji A. Experimental investigation of waste glass powder as the partial replacement of cement in concrete production. Proceedings of the International Conference on Advances in Engineering & Technology.

[52] Hussain G., Verma G. (2016). Experimental investigation on glass powder as partial replacement of cement for M-30 concrete. Int. J. Sci. Res. Sci. Eng. Technol..

[53] Nassar R.-U., Soroushian P. (2011). Field investigation of concrete incorporating milled waste glass. J. Solid Waste Technol. Manag..

[54] Metwally I.M. (2007). Investigations on the Performance of Concrete Made with Blended Finely Milled Waste Glass. Adv. Struct. Eng..

[55] Ibrahim K.I.M. (2021). Recycled waste glass powder as a partial replacement of cement in concrete containing silica fume and fly ash. Constr. Build. Mater..

[56] Islam G.S., Rahman M., Kazi N. (2017). Waste glass powder as partial replacement of cement for sustainable concrete practice. Int. J. Sustain. Built Environ..

[57] Tamanna N., Tuladhar R. (2020). Sustainable Use of Recycled Glass Powder as Cement Replacement in Concrete. Open Waste Manag. J..

[58] Ali I., IFTM University (2015). Behavior of Concrete by using Waste Glass Powder and Fly Ash as a Partial Replacement of Cement. Int. J. Eng. Res..

[59] Kumar V., Sood H. (2017). Effect of Waste Glass Powder in Concrete by Partial Replacement of Cement. Int. J. Civ. Eng..

[60] Bhagyasri T., Prabhavathi U., Vidya N. (2016). Role of Glass Powder in Mechanical strength of concrete. Int. J. Adv. Mech. Civ. Eng..

[61] Patil B.H. (2020). Utilization of Waste Glass Powder as a Replacement of Cement in Concrete. Int. J. Res. Eng. Sci. Manag..

[62] Hussain M.V., Chandak R. (2015). Strength properties of concrete containing waste glass powder. Int. J. Eng. Res. Appl..

[63] Al-Zubaid A.B., Shabeeb K.M., Ali A.I. (2017). Study The Effect of Recycled Glass on The Mechanical Properties of Green Concrete. Energy Procedia.

[64] Ramdani S., Guettala A., Benmalek M., Aguiar J.B. (2019). Physical and mechanical performance of concrete made with waste rubber aggregate, glass powder and silica sand powder. J. Build. Eng..

[65] Nergis D.D.B., Vizureanu P., Ardelean I., Sandu A.V., Corbu O.C., Matei E. (2020). Revealing the Influence of Microparticles on Geopolymers’ Synthesis and Porosity. Materials.

[66] Hao D.L.C., Razak R.A., Kheimi M., Yahya Z., Abdullah M.M.A.B., Nergis D.D.B., Fansuri H., Ediati R., Mohamed R., Abdullah A. (2022). Artificial Lightweight Aggregates Made from Pozzolanic Material: A Review on the Method, Physical and Mechanical Properties, Thermal and Microstructure. Materials.

[67] Kim J., Yi C., Zi G. (2015). Waste glass sludge as a partial cement replacement in mortar. Constr. Build. Mater..

[68] Najad A.A.A.-J., Kareem J.H., Azline N., Ostovar N. (2019). Waste glass as partial replacement in cement—A review. IOP Conf. Series Earth Environ. Sci..

[69] Ansari W.S. (2019). Porosity analysis using Image J. Proceedings of the 8th Graduate-Student Forum on Building Materials.

